# ECMOLIFE intra-hospital transport in life-saving for pulmonary vein obstruction

**DOI:** 10.1186/s40792-023-01702-y

**Published:** 2023-06-21

**Authors:** Ignazio Condello, Giuseppe Nasso, Gaetano Contegiacomo, Carlo Solimando, Giuseppe Balducci, Domenico Scaringi, Pasquale D’Alessandro, Giuseppe Speziale

**Affiliations:** 1 Department of Cardiac Surgery, Anthea Hospital, GVM Care & Research, Perfusion Service, Via Camillo Rosalba 35/37, 70124 Bari, Italy; 2 Department of Interventional Cardiology, Anthea Hospital, GVM Care & Research, Bari, Italy

**Keywords:** Pulmonary vein obstruction, Extracorporeal membrane oxygenation, Minimally invasive mitral valve repair, Minimally invasive cardiac surgery, Right heart failure, Respiratory failure

## Abstract

**Background:**

Transport with extracorporeal membrane oxygenation (ECMO) in the hospital setting can become a challenge as well as in the out-of-hospital setting. In particular, the management of intra-hospital transport with ECMO support of the critically ill patient foresees his shift from the intensive care to the diagnostic areas, from the diagnostic areas to the interventional and surgical areas.

**Case presentation:**

In this context, we present a life-saving transport case with the veno-venous (VV) configuration of the ECMOLIFE Eurosets system, for right heart and respiratory failure in a 54-year-old woman, due to thrombosed obstruction of the right superior pulmonary vein, following mitral valve repair surgery in minimally invasive approach in a patient already operated on for complex congenital heart disease. After stabilizing the vital parameters with Veno-venous ECMO for 19 h, the patient was transported to hemodynamics for angiography of the pulmonary vessels, where the diagnosis of obstruction of the pulmonary venous return was made. Subsequently, the patient was brought back to the operating room for a procedure of unblocking the right superior pulmonary vein using a minimally invasive approach, passing from the ECMO to the support in extracorporeal circulation.

**Conclusions:**

The transportable ECMOLIFE Eurosets System was safe and effective during transport in maintaining the vital parameters of oxygenation and CO_2_ reuptake and systemic flow, allowing the patient to be mobilized for diagnostic tests instrumental to diagnosis. The patient was extubated 36 h after the surgical procedures and was discharged 10 days later from the hospital.

## Background

Extracorporeal membrane oxygenation (ECMO) is a form of prolonged mechanical cardiopulmonary supportive therapy implemented for the survival of patients with refractory cardiac and respiratory dysfunction [1]. Venovenous (VV) and venoarterial (VA) ECMO are both used to buy time in severe respiratory failure while VA ECMO also provides hemodynamic support [2]. In the life-saving management of hospital complications, using a portable ECMO console is crucial to ensure safe and easy movement between the critical, diagnostics and interventional areas [3]. In this context, we present a life-saving transport case with the veno-venous (VV) configuration of the ECMOLIFE Eurosets SPA Medolla Italy system, for right heart and respiratory failure in a woman patient, due to thrombosed obstruction of the right superior pulmonary vein after cardiac surgery procedure.

## Case presentation

**Fig. 1:**
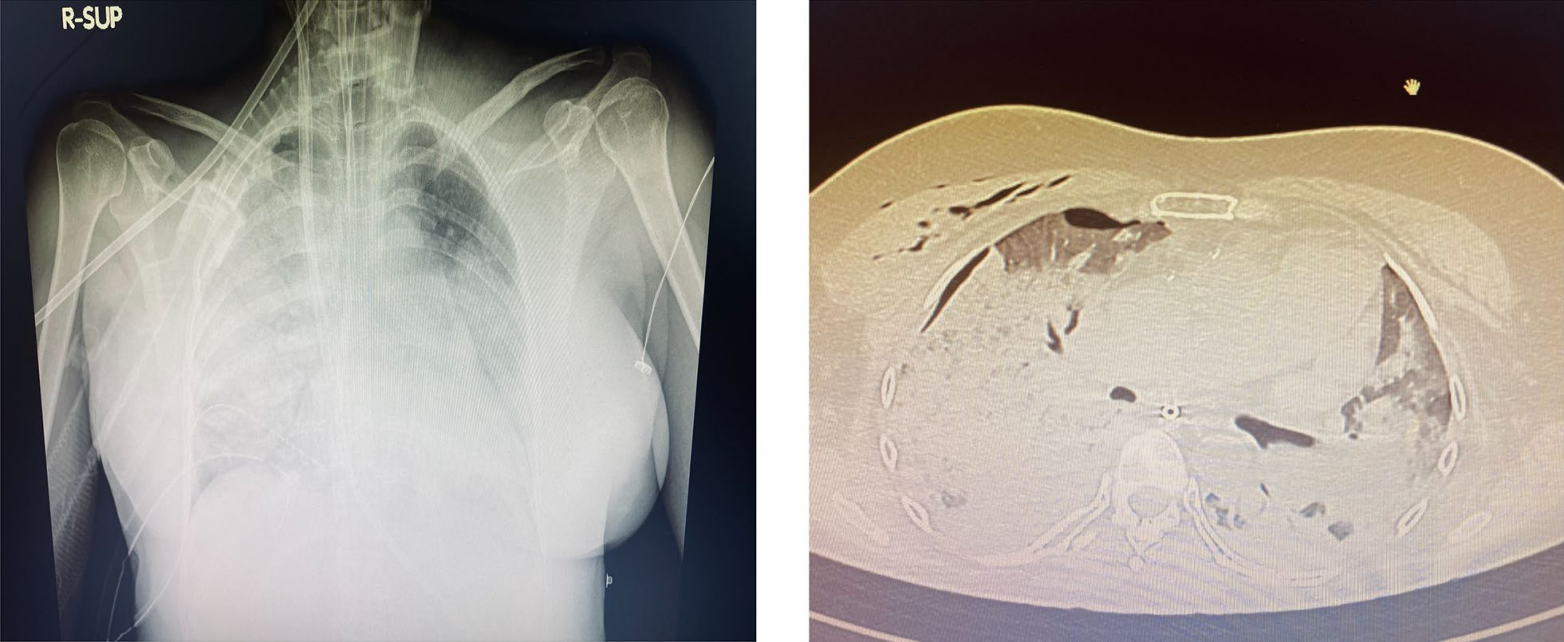
The chest X-ray and computerized axial tomography before the ECMO and surgical procedure

**Fig. 2:**
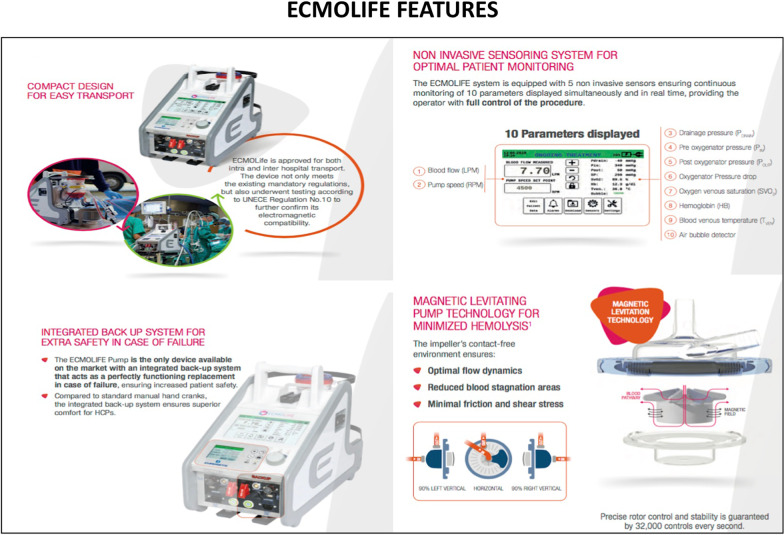
The ECMOLIFE console features

**Fig. 3:**
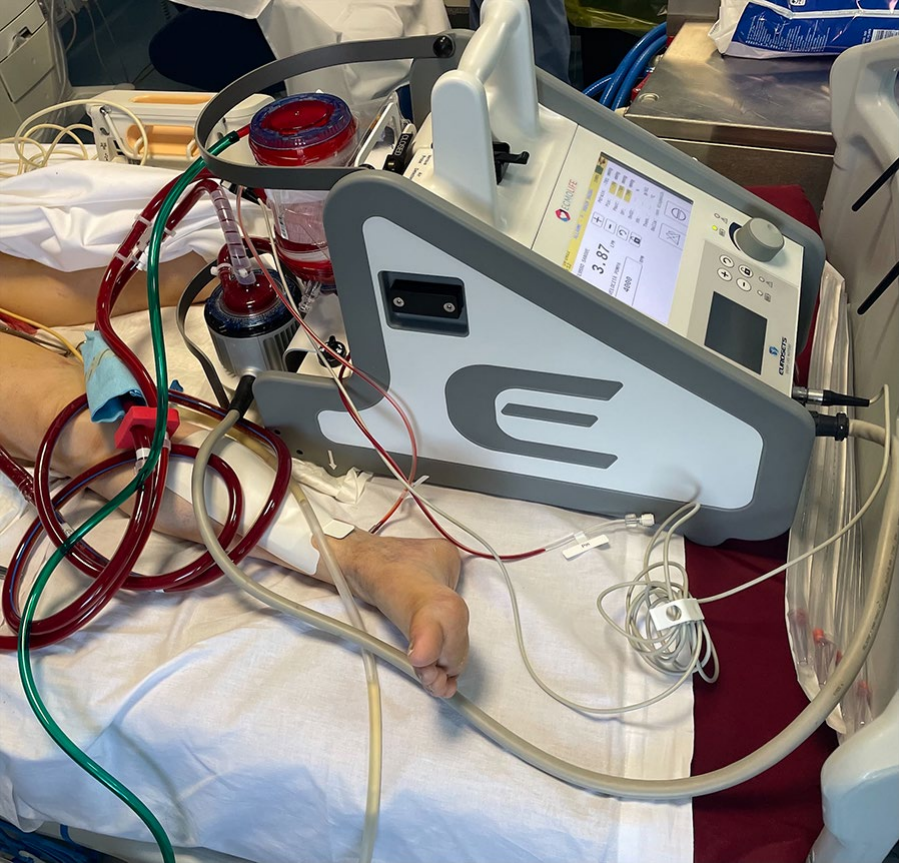
The ECMOLIFE during the transport

**Fig. 4:**
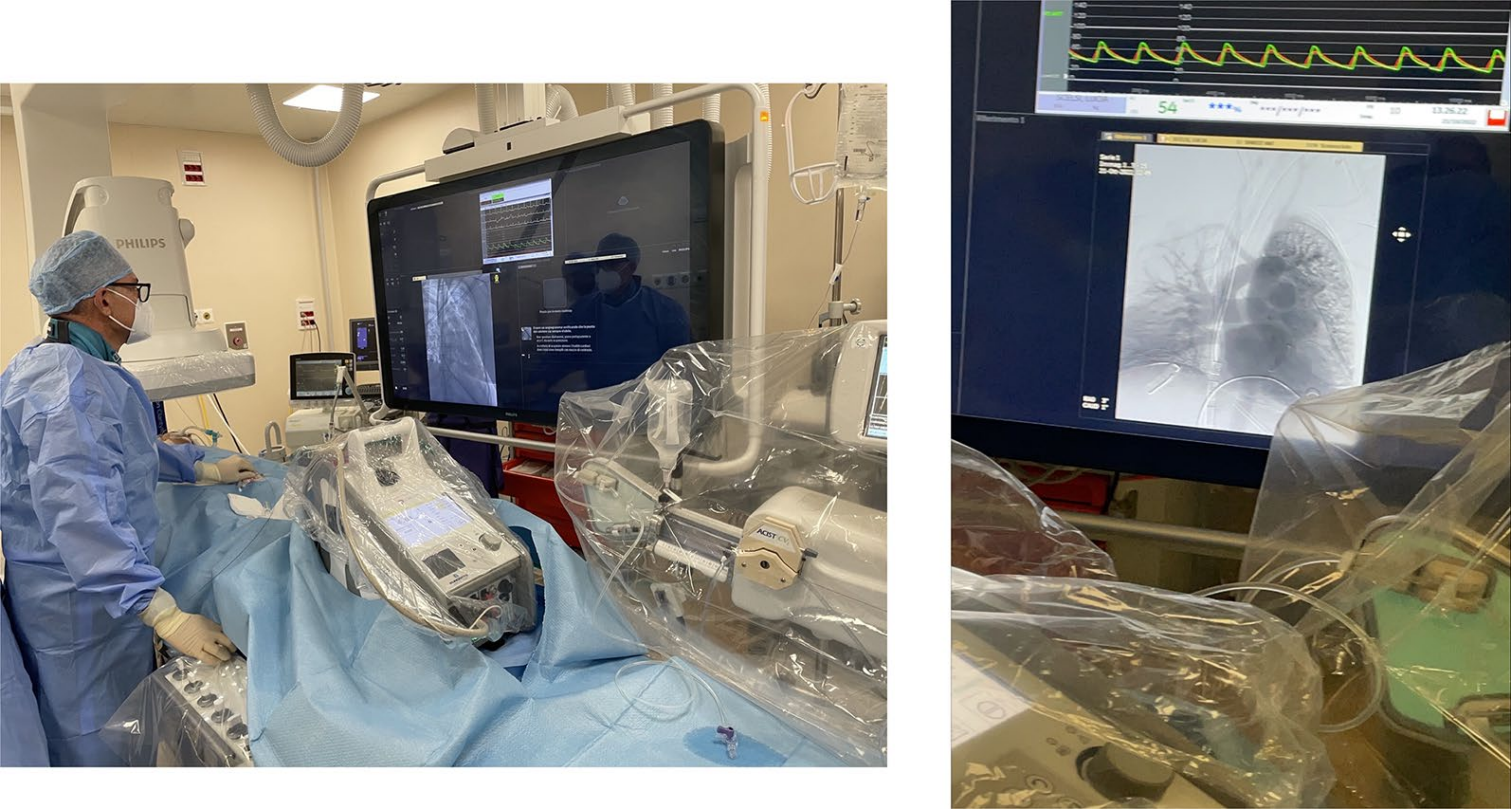
The angiography with hypoperfusion of the right apical lung and obstruction of the right superior pulmonary vein with temporary stop of the ECMO

**Fig. 5:**
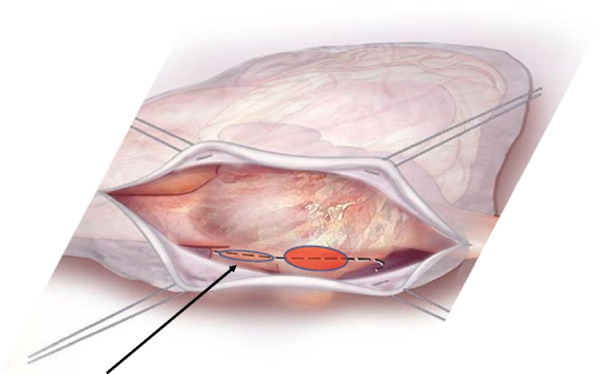
The reduction of the caliber of the right superior pulmonary vein outlet during the suture of the left atriotomy

**Fig. 6:**
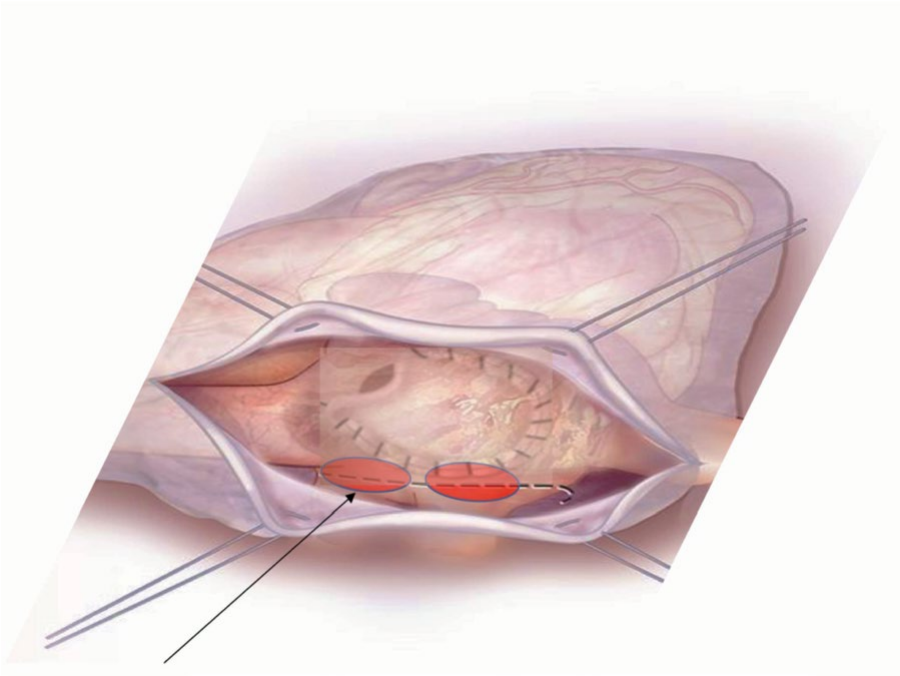
A pericardial patch was used to increase the surface area of the left atrium, to reduce the traction and the obstruction of right pulmonary vein

**Fig. 7:**
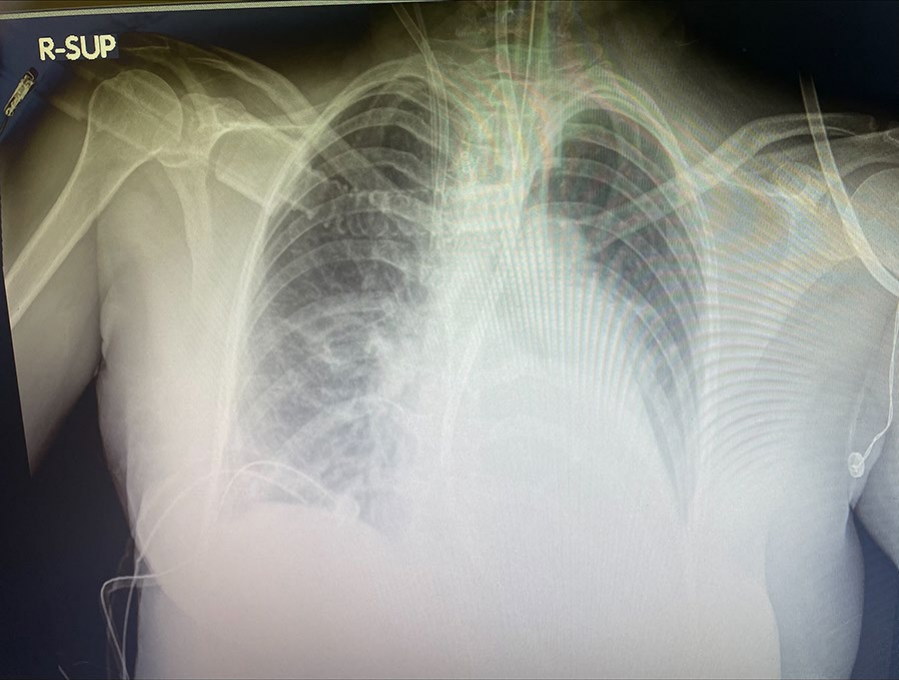
The chest X-ray after surgical procedure

A 54-year-old female patient already operated for closure of ostium primum and inter atrial defect at the age of 8 years-old, was treated for elective cardiac surgery procedure at our institution Anthea Hospital, GVM Care & Research, Bari, Italy, for mitral and tricuspid valve repair for severe mitral and tricuspid regurgitation and closure of residual inter atrial defect (ostium primum), through right anterior mini thoracotomy. The study was submitted and approved by institutional ethics committee. Our surgical approach for minimally invasive direct view during mitral and tricuspid surgery were described elsewhere, in this case reported a cardiopulmonary bypass time (CPB) of 85 min and cross clamp time 49 min [4, 5]. Immediately after mitral and tricuspid valve repair, transesophageal echocardiography showed a volume overload in terms of end-diastolic diameter of the right ventricle 60 mm, without alteration of the systolic function tricuspid annular plane systolic excursion (TAPSE) > 15 mm and absence of heart valve dysfunction after reparative surgery [6, 7]. However, pulmonary exchanges reported a worsening from baseline before surgery of arterial blood gas parameters PaO_2_ 87 mmHg with a SaO_2_ of 90%, a PaCO_2_ of 46 mmHg with a 100% FiO_2_ set on the ventilator with 87 mmHg PaO_2_/FiO_2_ (PF ratio), with a volume of 600 mL/min and a peak pressure of 65 cmH_2_O. After 24 h the procedure reported an abrupt worsening of the PF ratio of 65 mmHg prolonged for 3 h with a picture similar to ARDS in the chest radiograph (Fig. 1) which promptly indicated the ECMO VV. For the ECMO VV the ECMOLIFE (Euroset SPA, Medolla, Italy) console was used which uses a pump with magnetic levitation technology, the oxygenator used was in ALONE (Euroset SPA, Medolla, Italy) polymethylpentene fibers (Fig. 2) [8–10]. The coating of oxygenators and circuit was phosphorycoline Agile (Euroset SPA, Medolla, Italy). A 22 fr dual stage cannula (Livanova, London, UK) was used for the drainage of the inferior vena cava and a 17 fr Biomedicus (Medtronic, Minneapolis, USA) cannula for the re-delivery of oxygenated blood into the right jugular vein. A flow of 3.8 L/min was set in the ecmo set in relation to the cardiac index of the Swan Ganz catheter. Patient characteristics 56 kg and 165 cm. Anticoagulant therapy involved the administration of heparin in continuous infusion to maintain an activated clotting time (ACT) of 200 s and an activated partial thromboplastin time (aPTT) of 52 s. After the establishment of the ECMO, the lungs were managed with protective ventilation in terms of FiO_2_, volumes, positive end-expiratory pressure (PEEP), and ventilators per minute. At the blood gas test in radial artery during ECMO VV the patient reported 130 mmHg of PaO_2_ and 39 mmHg of PaCO_2_ (mean values). The patient was managed for 19 h in VV ECMO, because initially the case seemed like an ARDS; however, an alteration of the right upper pulmonary venous pattern emerged on the transesophageal ultrasound, giving indication for further angiographic investigation. The patient supported by ECMO VV was moved to interventional cardiology (Fig. 3) subjected to transcatheter angiography of the pulmonary circulation from the right to the left heart (antegrade) and retrograde sections of the left atrium. During the angiographic study to study the pulmonary circulation during the administration of the contrast medium, ventilation and the flow of ECMO support were suspended, in order not to alter the normal physiological flow (Fig. 4). The ECMOLIFE console was placed in the angiographic bed between the patient's legs, covered with sterile cellophane. The ACT was monitored during the procedure which was kept around 280 s. After angiography, the diagnosis was made of obstruction of the right superior pulmonary vein probably obstructed during the suture of the left atrium in the surgical phase (Fig. 5) with the lobe of the right lung hypoperfused by stasis resulting in respiratory failure. Subsequently the patient was brought to the operating room with the ECMO console placed on the patient's bed for the unblocking of the right pulmonary vein, through reintervention in a minimally invasive surgical approach through the previous right upper minithoracotomy to the 3rd intercostal space [5, 6]. The patient was switched from ECMO to cardiopulmonary bypass (CPB) and the ECMO circuit blood was returned the ECMO cannulas were used for drainage in CPB [9]. A clot was removed from the right superior pulmonary vein. probably the formation of the clot was given by the reduction of the lumen narrowing of the right upper pulmonary vein which was determined by the previous atrial suture, for this reason a pericardial patch was used for enlarged the left atrium during the suture to maintain the caliber of the right pulmonary vein (Fig. 6). The patients weaning from CPB did not require ECMO for a P/F ratio of 180 mmHg. The patient was extubated 36 h after the surgical procedures with an improved chest X-ray (Fig. 7) and was discharged 10 days later from the hospital, without complications.

## Discussion and conclusion

In patients with acute respiratory distress syndrome (ARDS), VV ECMO is recommended if the PF ratio is less than 50 mmHg for more than 3 h or less than 80 mmHg for more than 6 h, based on WHO guidelines. If the patient’s condition does not improve even after conventional management with optimal ICU care, e.g., mechanical ventilation (MV), prone positioning, ECMO should be recommended within 5 days from the initiation of invasive MV [11].

There are several consoles in the market validated for ECMO transport, such as Cardiohelp, Getinge, Rotaflow II Getinge and others, however ECMOLIFE Eurosets SPA, Medolla, Italy, is the first portable console with magnetic levitation technology with the aim of minimizing hemolytic damage. However, there are no data available in the literature on complications and mortality related to transport, often difficult to manage due to non-integrated devices in terms of circuit and console monitoring. The integrated technical characteristics of the ECMOLIFE system, in terms of monitoring and control of the inlet and outlet pressures of the magnetic levitation pump inlet oxygenator, the integrated monitoring of oxygenation parameters (SvO_2_), and the integrated circuit to the oxygenator on the machine have made transportation simple in our experience. ECMO remains a high-cost therapy with life-saving potential in a select group of critically ill patients. Given the level of experience required for the day-to-day care of these patients, it is preferable that ECMO candidates be transferred to a specialized referral center. Due to severe respiratory insufficiency, patient transfer without ECMO is generally considered too risky. In contrast, there are no data on the safety of inter-hospital ECMO transports. In a case series by Pedro Vitale Mendens et al. reported that the patients transported with ECMO support to a referral hospital in Brazil with a survival rate of 57% and no major complications or deaths during transport. The systematic literature review by Pedro Vitale Mendens et al. showed a pooled survival rate for adult and pediatric patients of nearly two-thirds, with only 2 deaths reported in this cohort of 1481 patients, and no other major adverse events resulting from the transport itself [6]. The data are compatible with the overall mortality reported in the latest publication of the Extracorporeal Life Support Organization (ELSO), in which the expected survival rate for extracorporeal venous support of adults was 58% [9]. Similarly, using the available published data, we found a 62% survival rate for adult patients transported during ECMO. For the pediatric population, our pooled analysis retrieved a survival rate of 68%, compared to 57% reported in the ELSO guidelines [11]. Thus, in this case series and in our overall analysis, we found no increase in mortality for ECMO support despite the need to refer the patient to a referral institution.

Concerns about the safety of transporting critically ill patients requiring extracorporeal support are an important issue to address considering the recent global increase in ECMO support [6, 7, 12]. In the Cesar study, patients randomized to the ECMO group were transferred to the referral center only after transport was deemed safe by the ECMO team, thereby delaying initiation of support. As reported in the text, patients were not transported during ECMO and, despite precautions, two deaths were reported during patient transfer. Similarly, in a previous publication of 158 infants accepted for ECMO initiation, Boedy et al. reported 18 (39.1%) transport-associated deaths [10]. Five newborns died while awaiting initiation of ECMO, and 13 died in transit without ECMO assistance or, after arriving moribund, before ECMO could be started. Considering that all of these deaths occurred before ECMO initiation, the authors concluded that there may be an undisclosed mortality associated with ECMO carriage that is generally excluded when we look exclusively at ECMO-supported patients [8, 9]. Thus, a strategy of early initiation of ECMO and patient transport during ECMO support may be safer than the use of conventional mechanical ventilation during referral transfer [10, 13]. Transport with ECMO in the hospital setting can become a challenge as well as in the out-of-hospital setting. In particular, the management of intra-hospital transport with ECMO support of the critically ill patient foresees his shift from the intensive care to the diagnostic areas, from the diagnostic areas to the interventional and surgical areas [1, 2]. In this context, having had a portable and versatile device for transport for ECMO assistance with ECMOLIFE (Euroset SPA, Medolla, Italy) was crucial for the maintenance of the patient’s vital functions and in carrying out movements aimed at carrying out instrumental diagnostics aimed at diagnosis.

## Data Availability

The datasets used and analyzed during the current study are available from the corresponding author on reasonable request.

## Abbreviations

ECMO: Extracorporeal membrane oxygenation
ARDS: Acute respiratory distress syndrome
CPB: Cardiopulmonary bypass
